# An 18-fluorodeoxyglucose-PET study in *SGCE* positive and negative myoclonus-dystonia

**DOI:** 10.1093/braincomms/fcag131

**Published:** 2026-05-06

**Authors:** Elze R Timmers, Henriëtte C J Dijkhuis, Ramesh S Marapin, Bauke M de Jong, Débora E Peretti, Jelle R Dalenberg, Marina A J Tijssen

**Affiliations:** Department of Neurology, University Medical Center Groningen, University of Groningen, Groningen 9713 GZ, the Netherlands; Expertise Center Movement Disorders Groningen, University Medical Center Groningen (UMCG), Groningen, Groningen 9713 GZ, The Netherlands; Department of Neurology, University Medical Center Groningen, University of Groningen, Groningen 9713 GZ, the Netherlands; Expertise Center Movement Disorders Groningen, University Medical Center Groningen (UMCG), Groningen, Groningen 9713 GZ, The Netherlands; Department of Neurology, University Medical Center Groningen, University of Groningen, Groningen 9713 GZ, the Netherlands; Expertise Center Movement Disorders Groningen, University Medical Center Groningen (UMCG), Groningen, Groningen 9713 GZ, The Netherlands; Department of Neurology, University Medical Center Groningen, University of Groningen, Groningen 9713 GZ, the Netherlands; Clinical Neuroimaging HUG, Hopitaux Universitaires de Genève, 1211 Genève, Switzerland; Expertise Center Movement Disorders Groningen, University Medical Center Groningen (UMCG), Groningen, Groningen 9713 GZ, The Netherlands; Department of Neurology, University Medical Center Groningen, University of Groningen, Groningen 9713 GZ, the Netherlands; Expertise Center Movement Disorders Groningen, University Medical Center Groningen (UMCG), Groningen, Groningen 9713 GZ, The Netherlands

**Keywords:** myoclonus-dystonia, [^18^F]FDG-PET, glucose metabolism

## Abstract

Myoclonus-dystonia is a hyperkinetic movement disorder, and approximately half of myoclonus-dystonia patients have a mutation in the *epsilon-sarcoglycan* (*SGCE*) gene, while the remaining cases often have undetermined causative genes. This study aims to assess brain metabolic function in myoclonus-dystonia patients with and without *SGCE* mutations and compare them with a control group. This study is part of the Next Move in Movement Disorders observational study. We included 23 myoclonus-dystonia patients (11 with *SGCE* mutations and 12 without) and 23 age-matched controls. Participants underwent 18-fluordeoxyglucose-PET and anatomical MRI scans. Data were analysed using a voxel-based analysis and a volume of interest (VOI)–based analysis. In the voxel-based analyses, trends towards differences in the supplementary motor area, cingulate gyrus, parietal and occipital lobe were found. When comparing mutation-positive with mutation-negative patients, trends towards differences in the parietal lobe and pre-central gyrus were detected. Symptom severity was correlated with changed metabolism in post-central and supramarginal gyrus, occipital and frontal lobe, cerebellum and caudate nucleus. In addition, VOI-based analyses showed statistically significant differences in the supplementary motor area comparing myoclonus-dystonia patients with controls. The identified trends of increased metabolism in the (pre)motor cortex areas fit the model of a more ‘excitable’ state with a lower activation threshold, possibly due to reduced inhibition from the cerebellum and striatum, regions in which we found a negative correlation between symptom severity and metabolism. Differences were also observed in sensory areas such as the parietal lobe and visual cortex. While the phenotype of *SGCE* mutation-positive and mutation-negative groups is similar, subtle differences suggest distinct endophenotypes.

## Teaser

This study examined brain metabolism in myoclonus-dystonia patients with and without *SGCE* mutations using 18-fluorodeoxyglucose-PET. Findings revealed altered metabolic activity in motor and sensory brain regions, with symptom severity linked to specific areas. Subtle metabolic differences suggest distinct endophenotypes between mutation-positive and mutation-negative patients, despite similar clinical presentations.

## Graphical Abstract

**Graphical Abstract:**
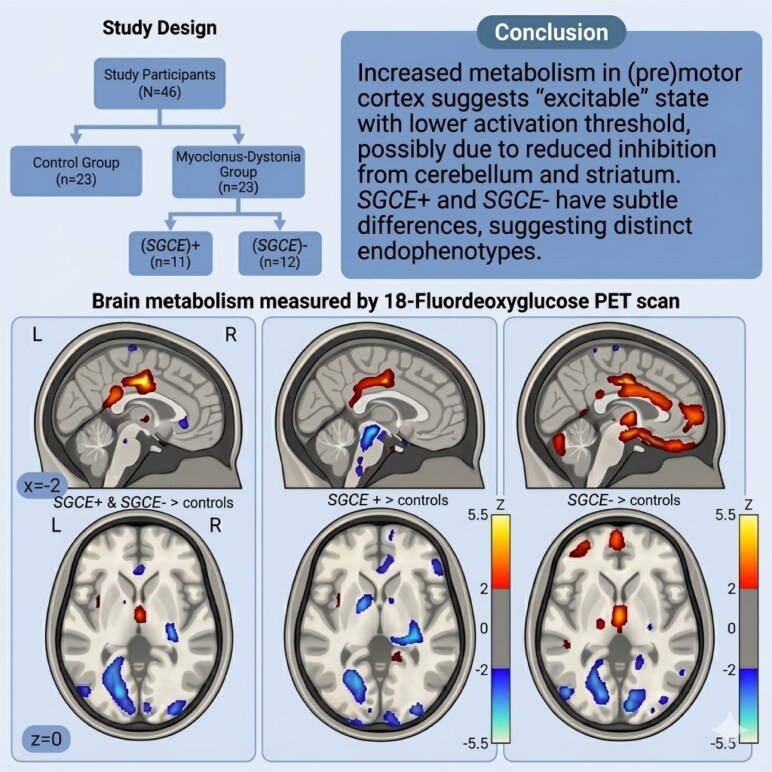

## Introduction

Myoclonus-dystonia (M-D) is a hyperkinetic movement disorder characterized by myoclonus and mild to moderate dystonia.^1^ Myoclonic symptoms mainly present as brief, ‘lightning-like’ jerks, mostly affecting the neck and arms.^2^ The dystonia is usually mild and manifests primarily in the neck and arms as cervical dystonia and writer’s cramp.^3^ About 30–50% of patients with M-D have a mutation in the *epsilon-sarcoglycan* (*SGCE*) gene, which has an autosomal dominantly inheritance pattern with maternal imprinting.^4–6^ In the remaining cases, the causative genes often remain undetermined; however, some other genes have been described (*ADCY5, KCTD17, CACNA1B* and *RELN*).^5,7^ The clinical phenotype of the *SGCE* gene mutation-negative patients usually resembles the mutation-positive patients, with mainly involvement of the upper body. However, increased rates of anxiety disorders and executive dysfunction have been described in the mutation-positive group.^8^ The question is whether to lump or to split these two groups. Roze *et al*.^9^ proposed to limit the term ‘M-D motor phenotype’ to patients only with an *SGCE-*like phenotype, including both genetic positive and negative patients as a phenotypic homogenous group.

SGCE is a membrane protein largely expressed in the CNS, particularly in the cerebellum.^10^ Although SGCE is causally linked to the M-D phenotype, the exact neurobiological mechanisms by which the *SGCE* mutation leads to the clinical manifestations of this disorder is incompletely understood. A wide range of research approaches (imaging,^11^ molecular^12^ and neurophysiological^13^) have been employed to investigate the pathophysiological mechanisms in M-D and implicated a primary dysfunction in the basal ganglia. However, more recent studies point towards a network disorder with the involvement of cerebello–thalamo–cortical (CTC) loop, possibly related to Purkinje cell disfunction.^9,10,14,15^ Only a few studies were performed in *SGCE* mutation-positive as well as mutation-negative patients. These showed that *SGCE* mutation-positive patients have increased cerebellar activity compared with mutation-negative patients.^16^ Overall, it is still unclear whether and to what extent the pathophysiology in *SGCE* mutation-positive and *SGCE* mutation-negative M-D is similar.

In this study, we aimed to assess brain metabolic function in patients with M-D, both in patients with and without a mutation in the *SGCE* gene and in a control group. All M-D patients presented with the typical M-D phenotype, with mainly upper body involvement for both dystonia and myoclonus. To this end, we used 18-fluordeoxyglucose-PET ([^18^F]FDG-PET), a non-invasive neuroimaging technique used to visualize regional cerebral glucose metabolism.^17^ Primarily, we expected to find abnormalities in the cerebellum, globus pallidus, thalamus and brainstem based on previous studies.^10^ In addition, based on the literature, we also expected to find changes in the parietal,^11,18^ somatosensory,^18^ motor,^13,18^ and ventromedial prefrontal cortex.^11^ To our knowledge, this is the first [^18^F]FDG-PET study that includes *SGCE* mutation-positive as well as *SGCE* mutation-negative patients with the typical M-D phenotype to determine similarities and differences in their pathophysiological mechanisms.

## Materials and methods

### Next move in movement disorders

This study is part of the Next Move in Movement Disorders (NEMO) study, an observational study that aims to develop a computer-aided diagnosis tool that helps specialists to improve the diagnosis, treatment and evaluation of hyperkinetic movement disorders.^19^ As part of the study, NEMO participants receive, among other tests, a [^18^F]FDG-PET and an anatomical MRI scan. The current study focuses on the [^18^F]FDG-PET and anatomical MRI scans of M-D patients and a control group without a movement disorder.

### Subjects

We included 23 M-D patients and 23 age-matched controls in the study. Since we included patients based on their clinical phenotype, M-D patients both with (*n* = 11) and without (*n* = 12) a mutation in the *SGCE* gene were enrolled. Patients were recruited using the Expertise Centre Movement Disorders Groningen hyperkinetic movement disorders database, from the University Medical Center Groningen (UMCG) movement disorders outpatient clinic, and we requested patients from other hospitals in the Netherlands through their physicians. *SGCE* mutation status was determined using targeted Sanger sequencing or next-generation sequencing panels for movement disorders. In the *SGCE*-negative group, additional testing was performed for known dystonia-related genes including TOR1A, THAP1, GCH1, CACNA1B and KCTD17, where clinically indicated (see Supplementary Table 1). Controls without a movement disorder were recruited through advertisements in the UMCG or were acquaintances of researchers involved in the NEMO study. All participants had to be ≥16 years of age. Patients or controls could not participate if they had other neurological conditions that induce movement disorders or if they had impaired hand or arm function. Controls were prohibited from participating if they were a first-degree family member of a patient with a hyperkinetic movement disorder. Furthermore, a silver allergy, an implanted pacemaker, pregnancy or currently breastfeeding were exclusion criteria. Specifically, participants were excluded if they would exceed the maximum annual radiation dose or had hyperglycaemia before the start of the [^18^F]FDG-PET scan (>7 mmol/l) and from the MRI scans if they had a contraindication for MRI scanning such as metal parts in the body and/or claustrophobia. Participants were recruited between February 2019 and January 2023. Healthy participants were matched with patients based on age and sex while prioritizing age over sex.

### Clinical assessment

All M-D participants had a clinically confirmed diagnosis made by an expert in the field of movement disorders. Clinical severity was assessed by two movement disorder researchers, using the global clinical impression severity (CGI-S) scale.^20^ To assess whether there was an indication of cognitive impairment, all participants filled out the Montreal Cognitive Assessment (MoCA). Furthermore, anxiety and depression were assessed using the Hospital Anxiety and Depression scale (HADS).^21^

### Standard protocol approvals and patient consents

Prior to participation, all participants gave written consent according to the World Medical Association Declaration of Helsinki 2008. The study was approved by the Medical Ethical Committee of the UMCG, METc code 2018/444.

### Imaging procedure and specifications

Details of the [^18^F]FDG-PET imaging protocol used in the NEMO study can be found in a previous publication.^19^ In summary, [^18^F]FDG-PET scans were acquired with a Siemens Biograph 40 mCT or 60 mCT PET/CT scanner at the Department of Nuclear Medicine and Molecular Imaging of the UMCG. Two hundred megabecquerels (199.6 ± 8.32) of [^18^F]FDG was injected through an intravenous catheter over a time period of roughly 30 s. Next, a buffered saline injection (±10 ml) was administered to flush out the cannula. A 20-min lasting (4 × 5) dynamic acquisition was performed 30 min after injection of the radiotracer.

List-mode data from all PET scans were reconstructed using 3D ordered-subset expectation maximization (3 iterations and 24 subsets), point spread function and time-of-flight corrections, resulting in 400 × 400 × 111 matrix, isotropic 2 mm voxels, smoothed with a 2-mm Gaussian filter at full width and half maximum.

MRI data were collected on a 3T Siemens Prisma scanner at the UMCG using a Siemens 64-channel head coil. High-resolution (MPRAGE) T1-weighted images were acquired using the following parameters: repetition time: 2300 ms; echo time: 2.98 ms; field of view: 256; FOV Phase: 93.8%; flip angle: 9°, 176 sagittal slices with 1 mm thickness.

### Image processing

Pre-processing was performed using a robust in-house pre-processing pipeline; for details, see Dalenberg *et al*.^19^ First, anatomical images were pre-processed in fMRIPrep version 20.2.0.^22^ Subsequently, Nipype (v1.8.3) was used for pre-processing PET images.^23^ The PET images were cropped using the autobox function from AFNI (v21.3.04).^24^ Next, we used HD-BET (v1.0) for brain extraction.^25^ Brain extracted images were then co-registered to the bias corrected anatomical images using Advanced Normalization Tools (v2.3.5).^26^ For the voxel-based analysis, linear transformations were merged with the normalization transformation matrix from fMRIprep and applied to the original PET image.

Two image analyses were performed. We first performed a voxel-based analysis using in-house developed scripts that make use of Nilearn 0.10.1 (https://nilearn.github.io/) running in Python 3.11.1.^27^ To this end, data were first scaled using robust scaling, which removes the median from each voxel and divides it by the interquartile range. As a result, uptake values represent the relative uptake compared to all other voxels in the brain. A general linear model framework was used to determine differences between experimental groups.

Second, we performed a regional analysis. For this analysis, image registration and data extraction were performed using PMOD software package (version 4.5). First, the T1 3D MRI was normalized to the Montreal Neurologic Institute spacing using tissue probability maps. Then, PET images were aligned to the individual MRI. The Automated Anatomical Labelling atlas was used to define anatomical brain volumes of interest (VOIs), a total of 122 regions, with white matter separated from cortical tissue. Robust scaling was applied to the images for intensity normalization with the same steps as in the voxel-based analysis. Regional values were then extracted for all brain regions. A general linear model framework was used to determine differences between experimental groups.

### Statistical analysis

Analyses on the demographics and clinical characteristics were performed in R version 4.4.1. Two sample *t*-tests and a Yates’s χ^2^ tests were used to test for group differences in demographics and clinical characteristics. To investigate group differences in brain metabolism, we performed voxel-based analyses implemented within a general linear model framework, adjusting for the possible confounders age and depression scores (anxiety scores were not taken into account as they strongly correlated with the depression scores) during all group comparisons.

For the voxel- and VOI-based analyses, we smoothed images with a 10-mm full width at half maximum kernel and performed a mass univariate regression analysis, using group as independent variable and age and depression scores as covariates. We investigated differences between healthy participants and M-D patients, differences between *SGCE* mutation-positive and mutation-negative patients, as well as differences between separate *SGCE* groups and healthy participants to better understand commonalities and differences between the patient subgroups. In the whole M-D group, we performed a mass univariate regression analysis, to assess the correlation of severity score with [^18^F]FDG-PET uptake. For enhanced interpretation of the voxel-based analysis, we will report results at multiple statistical thresholds (*P* < 0.001 uncorrected). Multiple comparison corrections were performed on a voxel level [*P*(false discovery rate (fdr)) < 0.05] and on a cluster level using threshold free cluster enhancement [*P*(tfce) < 0.5] with 10 000 permutations. In the tables, only clusters with a cluster size >5 voxels were shown; complete results can be found in Supplementary Tables 5 and 6. Furthermore, we will use a more liberal threshold (*P* < 0.05) for visualization purposes only.^28^ Data are visualized on the ICBM 152 Nonlinear atlas version 2009. For the VOI-based analysis, results were considered statistically significant when *P* < 0.05 uncorrected, and *P*(fdr) < 0.05.

## Results

### Demographic and clinical data

Initially, we included 25 M-D patients from the NEMO dataset, who received an [^18^F]FDG-PET and anatomical MRI scan. However, two *SGCE* mutation-positive patients were excluded prior to the analysis because of procedural errors concerning the saline injection dose. Finally, 23 M-D patients (mean age 35.35 ± 18.58 years) and 23 controls without M-D (mean 37.96 ± 18.82 years) remained. The M-D group consisted of 11 *SGCE* mutation-positive (mean 43.82 ± 19.71 years) and 12 *SGCE* mutation-negative patients (mean 27.6 ± 14.13 years). Table 1 shows the clinical characteristics of M-D patients and the subgroups of *SGCE* mutation-positive and mutation-negative patients and controls. In M-D patients, MoCA scores were significantly lower compared with controls. Ten M-D patients scored below the clinical cut-off value of 26, versus one participant in the control group. Anxiety and depression scores were higher in the patient group compared with the controls, with six patients having a score above the cut-off score of 7 (for both questionnaires), compared with one healthy participant who scored above the cut-off score for the depression scale.

**Table 1:** Clinical characteristics of healthy participants and M-D patients, and *SGCE* mutation-positive and mutation-negative M-D patients

	*HP (n = 23)*	*M-D (n = 23)*	*P*-value*^a^*	*SGCE mutation-positive (n = 11)*	*SGCE mutation-negative (n = 12)*	*P*-value *^a^*
*Age (range)*	38 (20–73)	35 (17–71)	0.64	44 (18–71)	28 (17–59)	<0.05
*Sex F/M*	9/14	5/18	0.20	0/11	5/7	<0.05
*CGI score (SD)*		3.40 ± 0.78		3.67 ± 0.78	3.15 ± 0.70	0.13
*Age of onset*		6 (0–36)		5 (0–13)	10 (0–36)	0.15
*MoCA*	28.50 ± 1.76^b^	26.22 ± 2.04	<0.001	25.73 ± 2.01	26.67 ± 2.06	0.28
*HADS anxiety*	2.47 ± 2.06^c^	6.78 ± 4.87	<0.001	7.36 ± 4.46	6.25 ± 5.36	0.60
*HADS depression*	1.37 ± 2.29^c^	3.78 ± 3.56	<0.05	4.00 ± 3.63	3.58 ± 3.63	0.79

HP, healthy participant; F, female; M, male.

^a^Mann–Whitney U-test for age (of onset), χ^2^ test for sex, *t*-tests for CGI-S score, MoCA and HADS.

^b^Five missing values.

^c^Four missing values.

### Metabolic differences between myoclonus-dystonia patients and healthy participants

**Figure 1:**
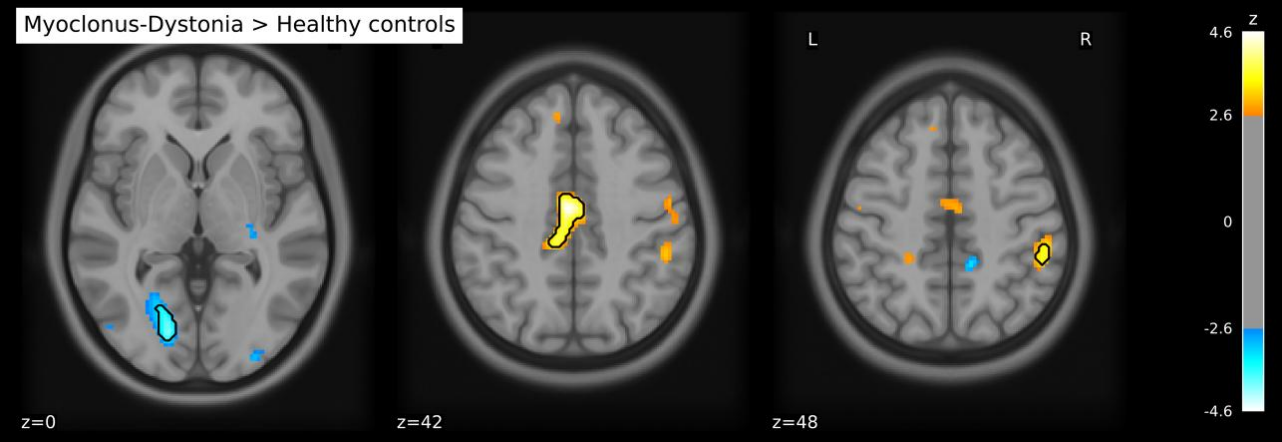
**Metabolic differences between M-D patients and healthy participants.** Voxel clusters showing differences in metabolism between M-D patients and healthy participants. Red and blue clusters indicate increased and decreased metabolism in M-D patients compared with healthy participants, respectively. Results are based on mass univariate regression analysis, using group as independent variable and age and depression scores as covariates. Results are displayed at a level *P* < 0.05 uncorrected for visual purposes; outlines show differences with a statistical threshold of *P* < 0.001 uncorrected. The colour bar represents *Z*-values.

The voxel-based analysis (Table 2; Fig. 1) revealed that, compared with controls, M-D patients showed trends towards increased metabolic activity in the left middle cingulate gyrus, left supplementary motor area and right inferior parietal lobe (all *P* < 0.001 uncorrected). Decreased activity was found in the left lingual gyrus and left frontal superior orbital gyrus (both *P* < 0.001 uncorrected). None of the found differences were statistically significant after FDR correction for multiple comparisons.

**Table 2:** Differences in brain metabolism between M-D patients and healthy participants

	Montreal neurological institute (MNI) coordinates					
Brain region	*x*	*y*	*z*	*z*_max_	Cluster size (mm^3^)^a^	*P* ^b,c^
**Metabolic increases in M**-**D patients**						
**Left middle cingulate gyrus**	−2	−10	42	4.58	1344	<0.001
	−8	−28	42	3.78		
**Right inferior parietal lobe**	52	−38	48	3.55	200	<0.001
**Left supplementary motor area**	−12	0	66	3.41	40	<0.001
**Metabolic decreases in M**-**D patients**						
**Left lingual gyrus**	−16	−78	0	−4.02	520	<0.001
**Left frontal superior orbital gyrus**	−22	48	−12	−3.38	64	<0.001

Reported *z*_max_ values have been adjusted for age and depression scores.

^a^Based on *P* < 0.001 uncorrected.

^b^Uncorrected for multiple comparison.

^c^All results were not statistically significant after correction for multiple comparisons using either FDR or TFCE.

**Figure 2:**
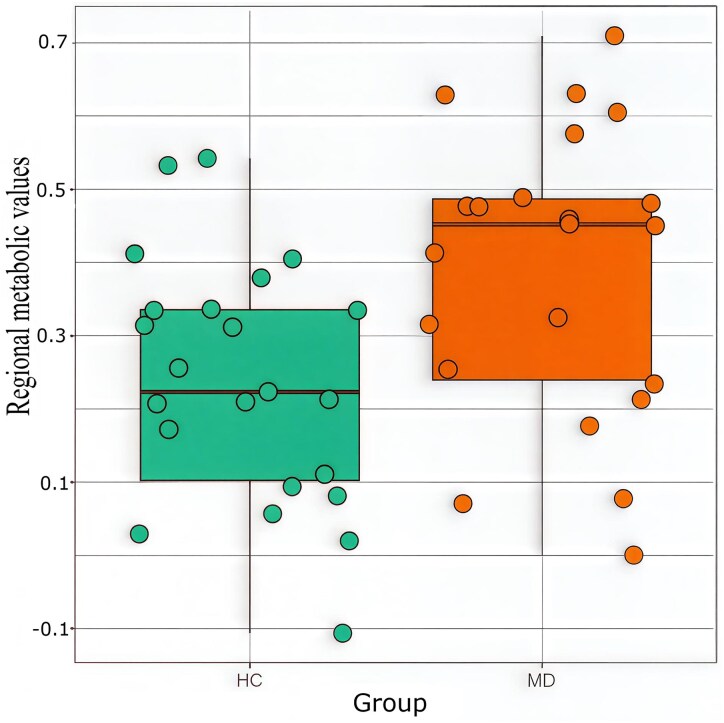
**Metabolic differences between M-D patients and healthy participants in the left supplementary motor area.** Boxplot representing the distribution of regional metabolic values for HCs (green) and M-D patients (orange) in the left supplemental motor area. Circles represent values for individuals separately.

The VOI-based analysis showed a significant increase in M-D patients compared with healthy control (HC) in the left supplementary motor area (*P* < 0.001 uncorrected), Left medial superior frontal cortex (*P* = 0.01 uncorrected), Vermis 10 (*P* = 0.01 uncorrected) and right middle cingulate cortex (*P* = 0.04 uncorrected), while a significant decrease was observed in the left superior temporal lobe (*P* = 0.04 uncorrected) (Supplementary Table 4). After correction for multiple comparisons, only the left supplementary motor area remained significant. Figure 2 displays regional values for the left supplementary motor area.

### Metabolic differences between *SGCE* mutation-positive and *SGCE* mutation-negative myoclonus-dystonia patients

**Figure 3:**
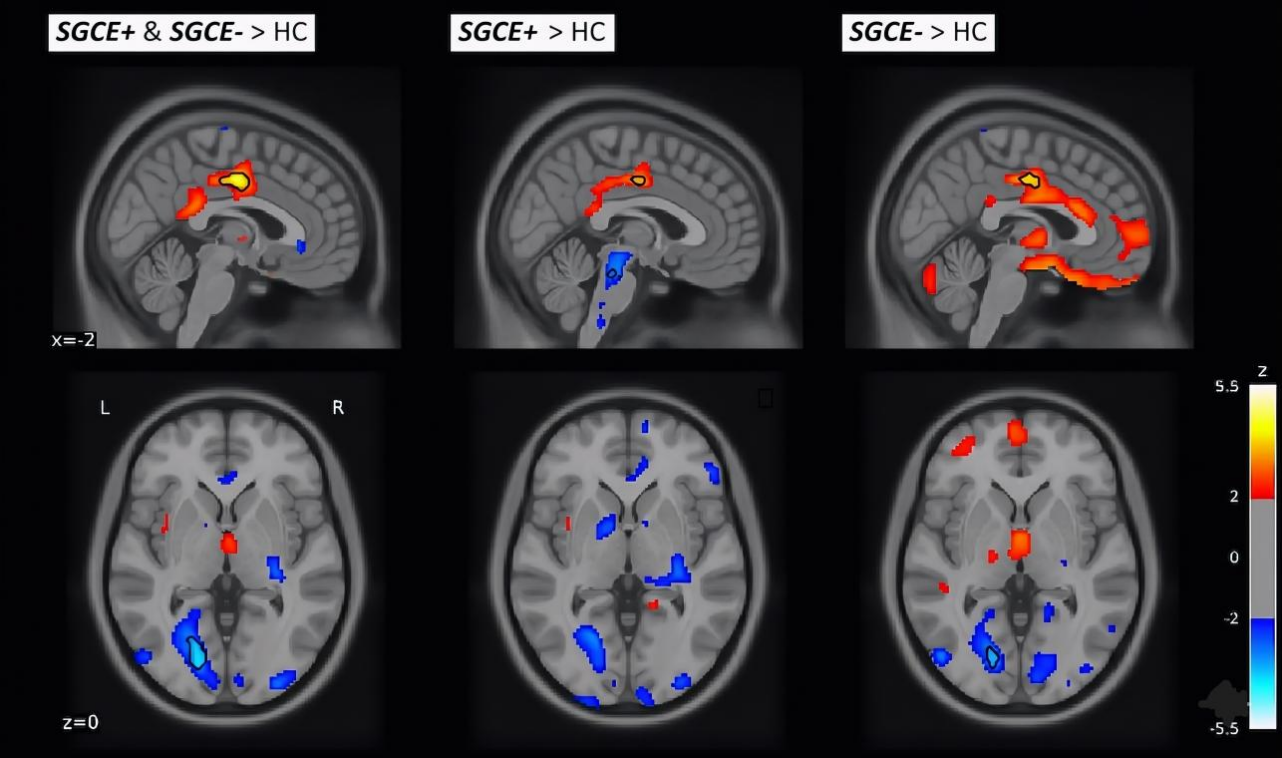
**Metabolic differences between all M-D patients, SGCE mutation-positive and mutation-negative patients versus healthy participants.** Voxel clusters showing correlations between metabolic change and severity of M-D. Results are based on mass univariate regression analysis, using group as independent variable and age and depression scores as covariates. Results are displayed at a level *P* < 0.05 uncorrected for visual purposes; outlines show differences with a statistical threshold of *P* < 0.001 uncorrected. The black outlines show significant differences with a statistical threshold of *P* < 0.001 uncorrected. The colour bar represents *Z*-values.

The voxel-based analysis (Table 3; Supplementary Fig. 1) revealed that, compared with *SGCE* mutation-negative patients, *SGCE* mutation-positive patients showed trends towards increased metabolic activity in the right and left precuneus, right inferior parietal lobule, right pre-central gyrus and the right lingual gyrus (*P* < 0.001 uncorrected). None of the found differences were statistically significant after FDR correction for multiple comparisons. These results indicate some differences between *SGCE* mutation-positive and mutation-negative patients. If we compare each subgroup separately with the control group without M-D, both groups show similar pathophysiological differences, with more profound differences in the orbital frontal lobe in the *SGCE* mutation-negative group and differences in the brainstem in the *SGCE* mutation-positive group (Fig. 3; Supplementary Tables 2 and 3).

**Table 3:** Clusters with increased brain metabolism in *SGCE* mutation-positive compared with *SGCE* mutation-negative M-D patients (no decreases were found)

	MNI coordinates				Cluster size (mm^3^)^a^	
Brain region	*x*	*y*	*z*	*z*_max_		*P* ^b,c^
**Right precuneus**	8	−48	16	4.56	1616	<0.001
	24	−54	22	3.82		
**Left precuneus**	−8	−62	34	3.72	360	<0.001
**Left precuneus**	−10	−58	16	3.60	48	<0.001
**Right inferior parietal lobule**	50	−52	42	3.58	88	<0.001
**Right precuneus**	16	−64	28	3.58	128	<0.001
**Right pre**-**central gyrus**	20	−28	66	3.50	40	<0.001
**Right lingual gyrus**	16	−44	0	3.38	56	<0.001

Reported *z*_max_ values have been adjusted for age and depression scores.

^a^Based on *P* < 0.001 uncorrected. Only clusters with a minimum size of 40 mm^3^ (=5 voxels) are reported; complete data can be found in Supplementary Table 5.

^b^Uncorrected for multiple comparisons.

^c^All results were not statistically significant after correction for multiple comparisons using either FDR or TFCE.

VOI-based analysis showed no significant differences between *SGCE* mutation-negative and *SGCE* mutation-positive patients. Comparing *SGCE* mutation-positive group separately with the HC individuals, a significant increase in FDG values was found in the left supplementary motor area (*P* < 0.01 uncorrected), Vermis 10 (*P* = 0.01 uncorrected), left medial superior frontal cortex (*P* = 0.01 uncorrected), right cerebellum III (*P* = 0.04 uncorrected), right supramarginal gyrus (*P* = 0.05 uncorrected) and right middle cingulum (*P* = 0.05 uncorrected). No significant decreases were found. After correction for multiple comparisons, only the left supplementary motor area remained significant *P*(fdr) < 0.05. Comparing *SGCE* mutation-negative patients with HC, a significant increase in patients was found in the left supplementary motor area (*P* = 0.03 uncorrected), and a decrease was found in the left superior temporal pole (*P* = 0.03 uncorrected). None of these differences were statistically significant after FDR correction for multiple comparisons.

### Correlation between brain metabolism and severity scores in myoclonus-dystonia patients

**Figure 4:**
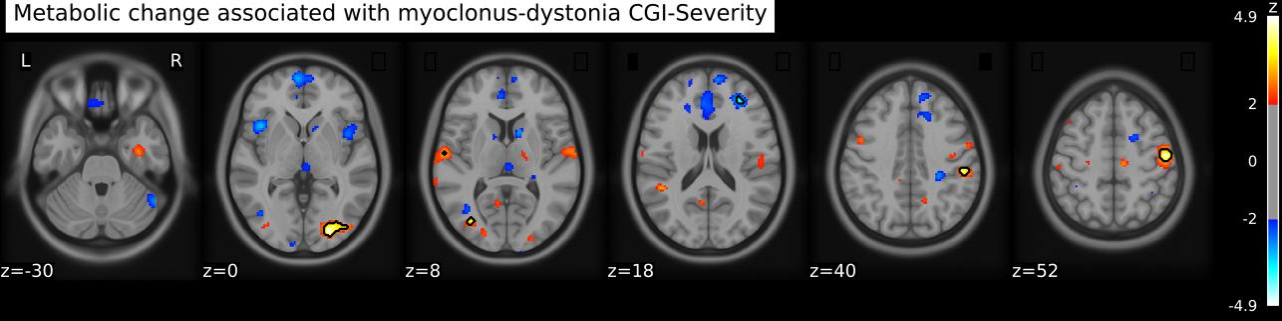
**FDG uptake correlated with M-D CGI severity.** Voxel clusters showing correlations between FDG uptake and CGI severity of M-D. Results are based on mass univariate regression analysis, using group as independent variable and age and depression scores as covariates. Results are displayed at a level *P* < 0.05 uncorrected for visual purposes; outlines show differences with a statistical threshold of *P* < 0.001 uncorrected. The colour bar represents *Z*-values.

FDG uptake increases with severity in the right fusiform, post-central, supramarginal gyrus and right middle occipital (*P* < 0.001 uncorrected) (Fig. 4; Table 4). FDG uptake had a negative correlation with severity in the right frontal lobe, cerebellum and caudate nucleus (*P* < 0.001 uncorrected). Scatter plots depicting the relationship (i.e. partial correlations) between severity and brain metabolism are provided in Supplementary Fig. 2 to demonstrate that correlations are not driven by outliers.

**Table 4:** FDG uptake associated with M-D CGI severity

	MNI coordinates			*z*_max_		
Brain region	*x*	*y*	*z*		Cluster size (mm^3^)^a^	*P* ^b,c^
**Right fusiform gyrus**	26	−86	0	4.76	896	<0.001
**Right inferior occipital lobe**	36	−82	−4	4.01		
**Right post**-**central gyrus**	52	−12	52	4.65	600	<0.001
**Right supramarginal gyrus**	52	−28	40	4.26	232	<0.001
**Left middle occipital lobe**	−34	−76	8	3.95	72	<0.001
**Right middle frontal lobe**	30	42	18	−3.57	72	<0.001
**Right cerebellum** C**rus 1**	52	−54	−30	−3.55	64	<0.001
**Right caudate nucleus**	12	14	6	−3.47	40	<0.001

Reported *z*_max_ values have been adjusted for age and depression scores.

^a^Based on *P* < 0.001 uncorrected. Only clusters with a minimum size of 40 mm^3^ (=5 voxels) are reported; complete data can be found in Supplementary Table 6.

^b^Uncorrected for multiple comparisons.

^c^All results were not statistically significant after correction for multiple comparisons using either FDR or TFCE.

VOI-based analysis revealed an increased FDG uptake related to disease severity in the right rolandic operculum (*P* = 0.02 uncorrected). However, this difference did not remain significant after FDR correction for multiple comparisons.

## Discussion

We investigated changes in brain metabolism between patients with the typical *SGCE* motor M-D phenotype and controls, using [^18^F]FDG-PET. Both VOI-based and voxel-based analysis showed similar results, with a significant higher uptake of [^18^F]FDG in the supplementary motor area (SMA) in M-D patients compared with controls based on the VOI-based analysis. Both analyses showed trends of differences in brain metabolism in several areas—cingulate gyrus, frontal, parietal and occipital lobe—consistent with findings of previous studies.^11,13,18,29^ However, none of these differences reaches statistical significance. Surprisingly, we did not find profound differences in metabolism between the *SGCE* motor phenotype patients and controls in the basal ganglia, brainstem or cerebellum. However, severity of the motor symptoms was correlated to [^18^F]FDG uptake in the cerebellum and caudate nucleus. Comparing *SGCE* mutation-positive and *SGCE* mutation-negative patients, we detected differences in several areas, including the parietal and occipital lobe and pre-central gyrus. These results might indicate differences in cerebral function between *SGCE* mutation-positive and mutation-negative patients.

### Myoclonus-dystonia patients with *SGCE* motor phenotype compared with healthy controls

When interpreting the results of this study, it is important to mention that most of our findings were not statistically significant after correction for multiple comparisons. This could be due to the relatively small number of participants. Alternatively, it might be the result of the heterogenous nature of the group with both *SGCE* mutation-positive and *SGCE* mutation-negative patients.

Taking this limitation into account, one of the most striking findings of this study is that we did not find clear differences in the basal ganglia, brainstem or cerebellum when comparing all M-D patients with controls, despite previous studies suggesting that these areas are important in the pathophysiology of M-D.^9,10,15^ A lower metabolism in the brainstem was observed only when comparing *SGCE* mutation-positive patients with controls. In the last few years, it has become clearer that M-D is not the result of dysfunction of one specific brain area, but has to be considered a network disorder. The therapeutic effect of deep brain stimulation in the globus pallidus interna suggests a role for the striato–pallido–thalamo–cortical network,^30^ while imaging,^11^ molecular^12^ and neurophysiological^13^ studies point towards involvement of the CTC network. Although we did not find clear differences in the cerebellum, thalamus or basal ganglia, we did find differences in several cortical projection areas of these networks, such as the SMA and cingulate gyrus. These motor-related cortical regions showed increased metabolism in M-D patients. Furthermore, we found a negative correlation between the severity of M-D and uptake in the cerebellum and nucleus caudatus, supporting the role of these areas in M-D. The combination of this negative correlation in subcortical regions and M-D-related metabolic increases in cortical motor regions might point to a disbalance within the above referred networks with a disinhibitory effect on cortical projection regions. It is possible that due to the convergence of the involved networks in these projection areas, metabolic differences may only be detectable in these regions.

Although this is the first study to show altered metabolism of the SMA in M-D patients, it has been shown before in the metabolic patterns of patients with primary torsion dystonia and dopa-responsive dystonia.^31–33^ The SMA and cingulate gyrus are thought to play a critical role in planning and performance of complex motor tasks.^34–36^ A major contribution is particularly given to the temporal planning of movement. Increased activation of the cingulate cortex and more specifically the anterior cingulate cortex (ACC) has been reported before in M-D patients, consistent with our findings.^16,18^ Through its connections with the motor areas, the lateral prefrontal cortex and the thalamus and brainstem, the ACC plays an important role in the voluntary control of behaviour.^37,38^ It is suggested that dysfunction of the ACC is related to a higher susceptibility for dystonia.^39^ The higher metabolic activity that we found in these (pre-)motor areas might suggest ongoing (subtle) involuntary movements during the PET scan. Unfortunately, we did not have a surface EMG to correct for these possible movements. However, the myoclonus in M-D is considered to have a subcortical instead of a cortical generator.^14^ The increased cortical metabolism may also result from a disrupted or disinhibited motor state, where the threshold for movements is lower, leading to more involuntary movements.

M-D patients showed a trend towards increased metabolism in the parietal cortex and increased severity is correlated with increased FDG uptake. This is in line with earlier [^18^F]FDG-PET studies in patients with primary torsion dystonia and *SGCE* mutation-positive M-D patients.^11,39^ Additionally, an fMRI study in *SGCE* mutation-positive M-D patients also showed increased responsiveness of the parietal cortex during a finger tapping task.^18^ The parietal cortex plays a role in motor control and sensory-motor integration, and dysfunction is thought to be correlated with impaired sensory integration, as seen in dystonia.^39,40^

Furthermore, we found a trend towards decreased glucose metabolism in the left primary visual cortex (V1) in M-D patients. This is in line with a previous study in *SGCE* mutation-positive M-D patients that found a prolonged time to discern the minimal interval between two successive visual stimuli, which correlated with both myoclonus severity and cortical thickness of V1.^29^ This M-D-related impairment on the visual task specifically concerned temporal discrimination, without altered visuospatial discrimination. Moreover, visual network alterations have been described in primary dystonia.^41^ Based on these findings and our results one can postulate that altered visual sensory processing seems to play a role in M-D.

Several of the voxel-wise comparisons revealed unilateral trends, particularly in the left hemisphere. While we did not systematically assess motor symptom asymmetry in our cohort, clinical observations suggest that asymmetry is common in M-D, especially in dystonic features. Notably, the predominance of left-hemispheric findings may be influenced by the fact that most participants were right-handed, which could reflect lateralized motor control and sensorimotor integration. Future studies should include standardized assessments of motor asymmetry and handedness to better interpret lateralized metabolic patterns and their clinical relevance.

### *SGCE* mutation-positive and *SGCE* mutation-negative myoclonus-dystonia patients

When comparing mutation-positive patients with mutation-negative patients, trends towards differences in the parietal lobe and pre-central gyrus were found in the voxel-based analysis. This suggests differences between *SGCE* mutation-positive and mutation-negative patients. The aetiology of the mutation-negative group is considerably more heterogeneous, likely involving multiple, or other genes, which might explain the differences in results compared with the mutation-positive group. The overall group results appear to be primarily driven by the more homogenous *SGCE* mutation-positive subgroup. However, when comparing the mutation-positive and mutation-negative groups separately with controls, the results are largely similar, likely due to the predominantly similar phenotype. Nonetheless, there are some key differences: the *SGCE*-positive group, compared with controls, also shows changes in the brainstem, which aligns with findings from previous literature.^11^ A subtle difference in cingulate cortex involvement was noted between SGCE-positive and SGCE-negative patients, with a trend towards posterior cingulate cortex and anterior cingulate cortex (ACC) activity, respectively. However, this pattern was not consistently observed across the full volume and may be too limited to draw firm conclusions. Future studies with larger samples and targeted analyses are needed to explore this further. These findings highlight the distinct characteristics of *SGCE* mutation-positive patients and underscore the importance of considering genetic variability in understanding the pathophysiology of M-D.

This study is the first [^18^F]FDG-PET study to include both *SGCE* mutation-positive and mutation-negative patients, resulting in a relatively large sample size for a rare disease as M-D, especially compared with previous imaging studies with sample sizes around 12–13 patients.^11,18^ However, as mentioned before, the potential genetic heterogeneity within the mutation-negative group may have influenced the results. Additionally, the mutation-positive group consisted solely of males, which could have further impacted the results, because of the small groups we chose not to correct for this in the analysis. Despite this, our analysis accounted for age and psychiatric co-morbidity. The mutation-positive group had slightly, although not statistically different, higher values on the scales measuring depression and anxiety than the mutation-negative group and lower values measuring cognitive functioning (MOCA). The slightly lower scores on the MOCA that we found in the M-D group are in line with previous findings showing executive dysfunction in M-D patients.^8^ MoCA scores were not used as covariates due to incomplete data and their secondary relevance to our primary research question. These choices may have influenced the results and should be considered when interpreting the findings. Depression and anxiety are common non-motor symptoms in M-D and are considered part of the phenotype.^9^ Especially frontal areas, as well as the ACC, are thought to be involved in the pathophysiology of anxiety and depression.^42,43^ In this study we chose to correct for these non-motor symptoms to focus on the metabolic differences caused by the motor symptoms. The differences in the cingulate cortex and the correlations we observed between several frontal areas of M-D support the hypothesis of a shared pathophysiology of the non-motor and motor symptoms.

A limitation of this study is that participants were not instructed to abstain from medication or alcohol prior to scanning. This may have influenced FDG uptake, particularly in regions sensitive to pharmacological effects. Future studies should consider standardized pre-scan protocols to minimize such confounding factors. Standardized motor rating scales such as the unified Parkinson's disease rating scale and unified myoclonus rating scale were not available in our cohort, as they were not part of the NEMO study protocol.^44^ Instead, we used the CGI-S scale to assess overall clinical severity. While less specific, this global measure allowed us to explore symptom-related metabolic changes, which should be refined in future studies using dedicated motor scales.

A limitation of the VOI-based analysis is the relatively small sample size (46 individuals) compared to the large number of VOIs analysed (116 regions), which reduces statistical power. Larger cohorts or studies using a selected amount of VOIs could confirm our findings and allow more robust correction methods. While the current study focuses on group-level comparisons, future work will aim to incorporate inter-subject covariance network analyses to further explore the network-based mechanisms underlying M-D. Such approaches, including scaled subprofile model - principle component analyses, may offer complementary insights beyond the scope of the present manuscript.

## Conclusion

Our study identified trends of increased metabolism primarily in the (pre)motor cortex areas, including the SMA, cingulate gyrus and pre-central gyrus. This suggests a more ‘excitable’ state with a lower threshold for activation. This is possibly due to reduced inhibition from the cerebellum and striatum, despite that only a negative correlation between symptom severity and metabolism was found in these regions without significant metabolic change. Additionally, differences were observed in sensory areas such as the parietal lobe and, surprisingly, the visual cortex. Our results suggest that, in part, the pathophysiology of the mutation-positive and mutation-negative group is similar. However, subtle differences were noted when comparing *SGCE* mutation-positive and mutation-negative groups. This finding suggests that although the two groups overlap in motor phenotype, their endotypes may differ. In the future, research into the (genetic) origin of M-D in the *SGCE*-negative group is needed to provide greater insight into the pathophysiology of this group. Overall, our study highlights the complex interplay between motor and sensory regions in M-D, suggesting that both shared and distinct mechanisms contribute to the observed clinical phenotype.

## Supplementary Material

fcag131_Supplementary_Data

## Data Availability

The data that support the findings of this study are available upon reasonable request from the corresponding author. The code that was generated for data analysis can be found in the Supplementary material.
