# Supplementary material for: An 18-fluorodeoxyglucose-PET study in SGCE positive and negative myoclonus-dystonia
Source: Brain Commun. 2026 May 6;8(3):fcag131. doi: 10.1093/braincomms/fcag131 (PMC13184688; doi:10.1093/braincomms/fcag131)
Supplement: fcag131_Supplementary_Data [file fcag131_supplementary_data.pdf]

Supplementary figure 1

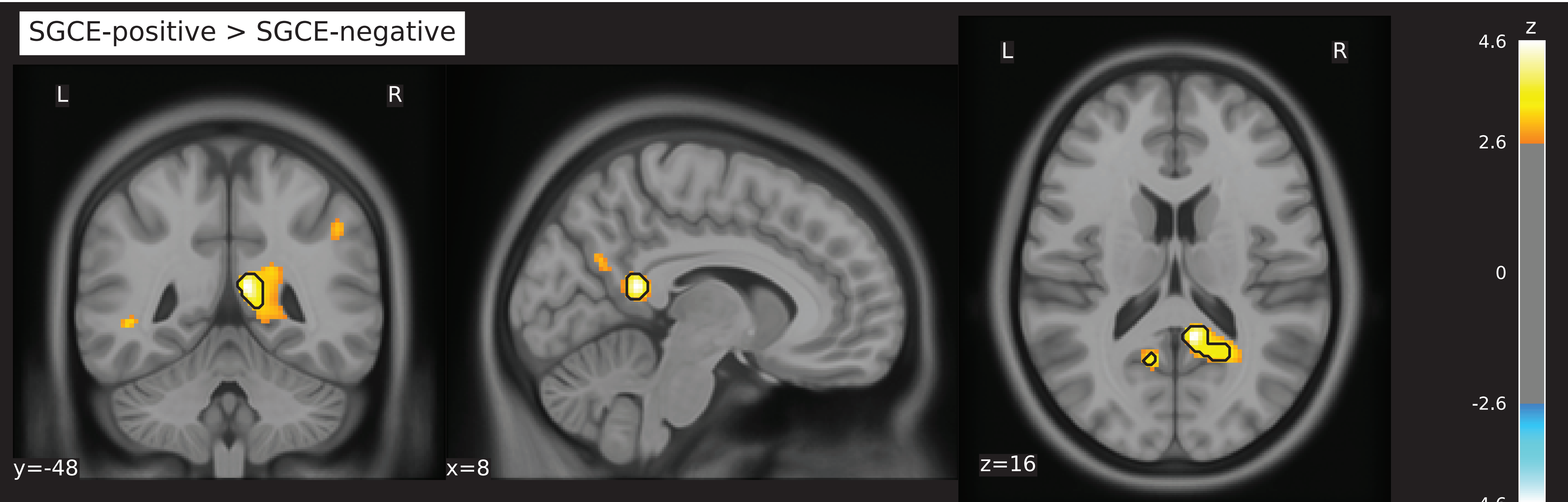

Voxel clusters showing differences in metabolism between SGCE positive and negative patients. Orange clusters indicate increased metabolism in SGCE mutation postive patients vs mutation negative patients. Results are based on mass univariate regression analysis, using group as a independent variable, and age and depression scores as covariates. Results are displayed at a level $p<0.05$ uncorrected for visual purposes, outlines show differences with a statistical treshold of $p<0.001$ uncorrected,

Supplementary figure 2

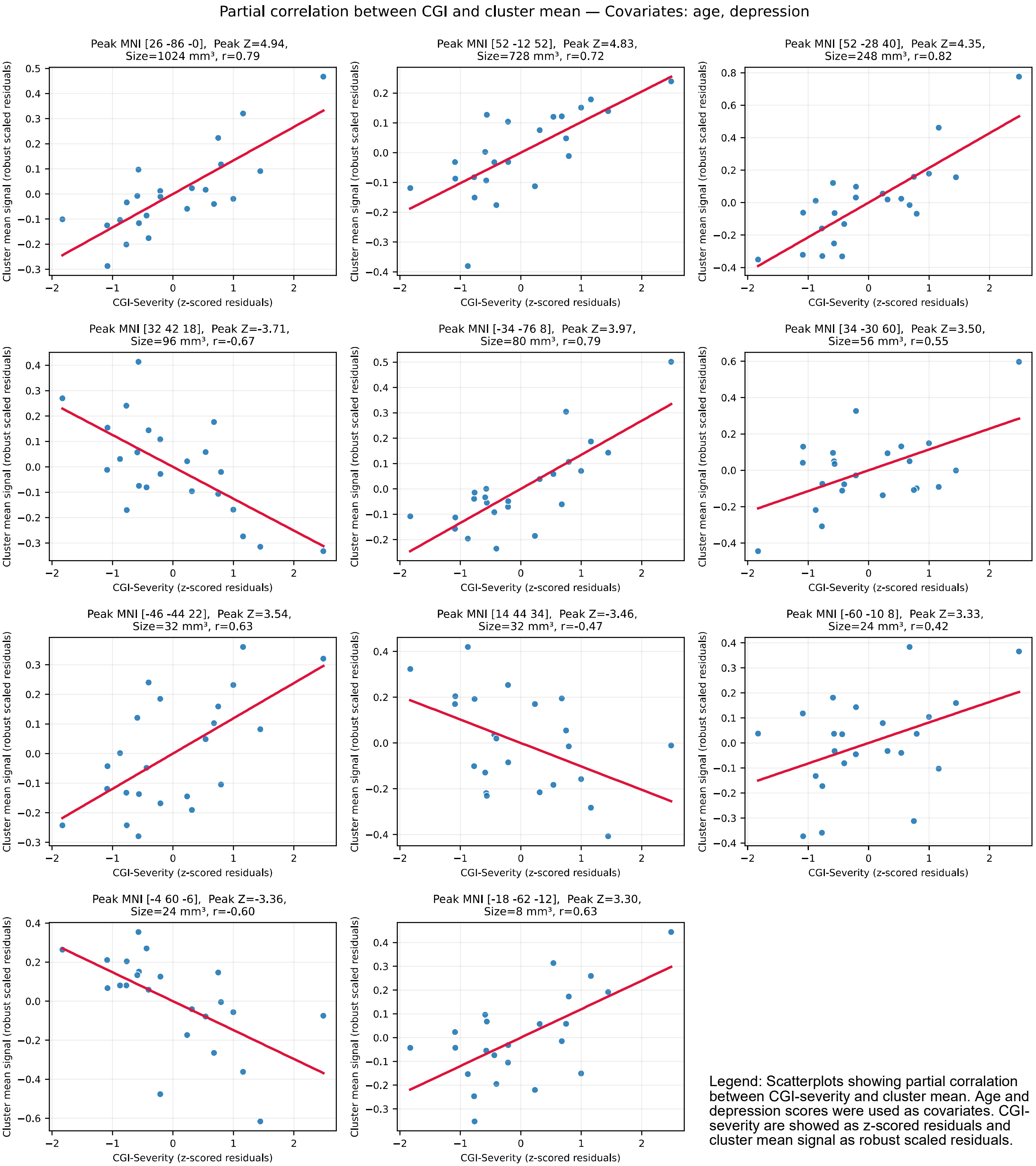

Legend: Scatterplots showing partial corralation between CGI-severity and cluster mean. Age and depression scores were used as covariates. CGI-severity are showed as z-scored residuals and cluster mean signal as robust scaled residuals.

Supplementary tables

Supplementary table 1 Details about genetic testing and etiology of *SGCE* mutation negative myoclonus dystonia patients

PATIENT	ETIOLOGY	GENETIC TESTING
1.	Genetic suspected	Whole exome sequencing with gene panel for movement disorders did not reveal pathogenic variant
2.	Genetic suspected	No analysis done
3.	Genetic suspected	No analysis done
4.	Genetic suspected	Whole exome sequencing with gene panel for movement disorders did not reveal pathogenic variant
5.	Genetic suspected	No analysis done
6.	Genetic suspected	Whole exome sequencing with gene panel for movement disorders did not reveal pathogenic variant
7.	Genetic suspected	Whole exome sequencing with gene panel for movement disorders did not reveal pathogenic variant
8.	Genetic suspected	Whole exome sequencing with gene panel for movement disorders did not reveal pathogenic variant
9.	Unknown	Unknown
10.	Unknown	Unknown
11.	Genetic suspected,	Unknown
12.	Uniparental disomie on chromosome 7	Whole exome sequencing with gene panel for movement disorders

Supplementary table 2: Differences in brain metabolism between *SGCE* mutation-positive M-D patients and healthy participants

	MNI coordinates						
**Brain region**	X	Y	Z	Z_{max}[a]	Cluster size (mm^3)[b]	P[c]	p(fdr)[d]
Left Supplementary motor area	-8	0	62	3.89	352	<0.05	n.s.
Left Supplementary motor area	-8	-18	62	3.82	176	<0.05	n.s.
Left precuneus	-12	-42	64	3.81	352	<0.05	n.s.
	-14	-54	64	3.64			
Left middle cingulum	-4	-10	42	3.72	160	<0.05	n.s.
Left middle cingulum	-10	-30	42	3.60	160	<0.05	n.s.
Left superior medial frontal lobe	-8	40	40	3.48	104	<0.05	n.s
	-8	34	46	3.48			
Left supplementary motor area	-8	16	58	3.46	32	<0.05	n.s.
Right supramarginal gyrus	58	-28	44	3.33	24	<0.05	n.s.
Right precentral gyrus	28	-22	64	3.30	8	<0.05	n.s.
Left cerebellum crus 1	-40	-84	-22	-3.67	688	<0.05	n.s.
	-20	-88	-16	-3.53			
Right medial orbital frontal lobe	12	64	-8	-3.53	48	<0.05	n.s.
Left pons	-6	-28	-22	-3.40	64	<0.05	n.s.
Left lingual gyrus	-20	-64	2	-3.30	8	<0.05	n.s.

Reported Zmax values have been adjusted for age and depression scores.

[a] SGCE positive > Healthy participant

[b] based on p<0.05 uncorrected

[c] uncorrected for multiple comparisons

[d] corrected for multiple comparisons

Supplementary table 3: Differences in brain metabolism between SGCE mutation-negative patients and healthy participants

	MNI coordinates						
**Brain region**	X	Y	Z	Z_{max}[a]	Cluster size (mm^3)[b]	P[c]	P(fdr)[d]
Left middle cingulum	-2	-10	42	4.01	336	<0.05	n.s.
Left superior temporal pole	-18	32	-28	3.98	280	<0.05	n.s.

Right inferior temporal lobe	28	10	-50	3.85	184	<0.05	n.s.
Right rectus	6	36	-30	3.78	448	<0.05	n.s.
Left superior orbital frontal lobe	-10	18	-26	3.70	672	<0.05	n.s.
Left para hippocampal lobe	-8	2	-18	3.68	304	<0.05	n.s.
Right inferior orbital frontal lobe	38	32	-20	3.56	176	<0.05	n.s.
Left inferior parietal lobe	-34	-46	52	3.46	64	<0.05	n.s.
Left superior orbital frontal lobe	-12	58	-28	3.37	32	<0.05	n.s.
Left rectus	-6	54	-30	3.35	8	<0.05	n.s.
Left superior orbital frontal lobe	-14	58	-26	3.35	8	<0.05	n.s.
Left inferior parietal lobe	-56	-22	40	3.34	16	<0.05	n.s.
Right middle cingulum	4	-8	28	3.34	32	<0.05	n.s.
Right superior orbital frontal lobe	16	30	-28	3.33	16	<0.05	n.s.
Left supplementary motor area	-16	-4	66	3.32	16	<0.05	n.s.
Left superior orbital frontal lobe	-8	50	-30	3.31	8	<0.05	n.s.
Right supramarginal lobe	42	-14	28	-3.76	216	<0.05	n.s.
Left lingual gyrus	-16	-78	-0	-3.56	152	<0.05	n.s.
Right precuneus	22	-62	22	-3.39	16	<0.05	n.s.
Right precuneus	20	-64	24	-3.36	8	<0.05	n.s.

Reported Zmax values have been adjusted for age and depression scores.

[a] *SGCE mutation-negative > Healthy participant*

[b] *based on p<0.05 uncorrected*

[c] *uncorrected for multiple comparisons*

[d] *corrected for multiple comparisons*

Supplementary table 4 FDG uptake per region in healthy controls and Myoclonus dystonia patients, based on VOI-analyses. Results were not corrected for age and depression.

Average and standard deviation (SD) values for all VOIs included in the analysis from the intensity normalised images. Effect size measured through Cohen's d is shown on the table, with significant values in bold as defined by the 95% confidence interval. Last column contains the percentage difference between Myoclonus-Dystonia (MD) patients and Healthy Controls (HC).

REGION	HC	MD	COHEN'S D	% DIFFERENCE
**PRECENTRAL_L**	0.39 (0.12)	0.47 (0.15)	-0.58	20.11
**PRECENTRAL_R**	0.34 (0.1)	0.4 (0.16)	-0.39	15.35
**FRONTAL_SUP_L**	0.34 (0.18)	0.43 (0.24)	-0.45	28.81
**FRONTAL_SUP_R**	0.22 (0.25)	0.32 (0.35)	-0.33	46.33
**FRONTAL_SUP_ORB_L**	0.38 (0.22)	0.42 (0.16)	-0.19	9.25
**FRONTAL_SUP_ORB_R**	0.28 (0.27)	0.35 (0.23)	-0.28	25.14

**FRONTAL_MID_L**	0.61 (0.16)	0.67 (0.15)	-0.38	9.96
**FRONTAL_MID_R**	0.5 (0.19)	0.53 (0.31)	-0.1	5.15
**FRONTAL_MID_ORB_L**	0.6 (0.27)	0.58 (0.17)	0.08	-2.86
**FRONTAL_MID_ORB_R**	0.4 (0.29)	0.51 (0.29)	-0.38	27.14
**FRONTAL_INF_OPER_L**	0.63 (0.15)	0.68 (0.14)	-0.37	8.46
**FRONTAL_INF_OPER_R**	0.61 (0.17)	0.6 (0.19)	0.09	-2.69
**FRONTAL_INF_TRI_L**	0.69 (0.17)	0.71 (0.14)	-0.14	3.1
**FRONTAL_INF_TRI_R**	0.58 (0.16)	0.57 (0.23)	0.04	-1.26
**FRONTAL_INF_ORB_L**	0.53 (0.18)	0.52 (0.18)	0.05	-1.68
**FRONTAL_INF_ORB_R**	0.47 (0.16)	0.51 (0.13)	-0.22	6.82
**ROLANDIC_OPER_L**	0.43 (0.11)	0.49 (0.1)	-0.47	11.72
**ROLANDIC_OPER_R**	0.39 (0.16)	0.4 (0.14)	-0.08	3.03
**SUPP_MOTOR_AREA_L**	0.24 (0.17)	0.39 (0.19)	**-0.82**	62.03
**SUPP_MOTOR_AREA_R**	0.28 (0.15)	0.35 (0.17)	-0.47	27.42
**OLFACTORY_L**	-0.09 (0.08)	-0.09 (0.12)	0.07	8.2
**OLFACTORY_R**	-0.05 (0.09)	-0.02 (0.1)	-0.3	-56.11
**FRONTAL_SUP_MEDIAL_L**	0.32 (0.17)	0.41 (0.12)	**-0.61**	27.89
**FRONTAL_SUP_MEDIAL_R**	0.28 (0.17)	0.33 (0.2)	-0.3	20.75
**FRONTAL_MED_ORB_L**	0.41 (0.11)	0.43 (0.11)	-0.18	4.99
**FRONTAL_MED_ORB_R**	0.28 (0.17)	0.31 (0.21)	-0.13	8.62
**RECTUS_L**	0.36 (0.09)	0.39 (0.11)	-0.29	7.55
**RECTUS_R**	0.31 (0.11)	0.32 (0.13)	-0.12	4.56
**INSULA_L**	0.25 (0.09)	0.27 (0.1)	-0.22	8.38
**INSULA_R**	0.26 (0.12)	0.28 (0.11)	-0.23	10.03
**CINGULUM_ANT_L**	0.13 (0.17)	0.14 (0.16)	-0.08	10.34
**CINGULUM_ANT_R**	0.11 (0.17)	0.11 (0.22)	0.02	-3.49
**CINGULUM_MID_L**	0.49 (0.09)	0.53 (0.1)	-0.39	7.6
**CINGULUM_MID_R**	0.54 (0.09)	0.58 (0.08)	-0.5	8.29
**CINGULUM_POST_L**	0.86 (0.16)	0.88 (0.25)	-0.11	2.68
**CINGULUM_POST_R**	0.72 (0.2)	0.72 (0.2)	-0.01	0.19
**HIPPOCAMPUS_L**	-0.36 (0.07)	-0.39 (0.07)	0.42	8.55

**HIPPOCAMPUS_R**	-0.41 (0.11)	-0.45 (0.1)	0.33	8.54
**PARAHIPPOCAMPUS_L**	-0.31 (0.07)	-0.33 (0.13)	0.2	7.02
**PARAHIPPOCAMPUS_R**	-0.19 (0.08)	-0.22 (0.07)	0.48	19.85
**AMYGDALA_L**	-0.41 (0.07)	-0.45 (0.12)	0.41	9.72
**AMYGDALA_R**	-0.46 (0.16)	-0.52 (0.1)	0.48	13.48
**CALCARINE_L**	0.55 (0.16)	0.52 (0.17)	0.19	-5.86
**CALCARINE_R**	0.63 (0.12)	0.61 (0.16)	0.09	-1.89
**CUNEUS_L**	0.39 (0.16)	0.44 (0.16)	-0.29	11.62
**CUNEUS_R**	0.37 (0.18)	0.43 (0.17)	-0.32	14.65
**LINGUAL_L**	0.35 (0.11)	0.33 (0.15)	0.19	-7.14
**LINGUAL_R**	0.3 (0.13)	0.3 (0.17)	-0.03	1.71
**OCCIPITAL_SUP_L**	0.24 (0.19)	0.29 (0.14)	-0.29	20.44
**OCCIPITAL_SUP_R**	0.29 (0.16)	0.3 (0.13)	-0.1	5.19
**OCCIPITAL_MID_L**	0.42 (0.15)	0.39 (0.13)	0.27	-9.12
**OCCIPITAL_MID_R**	0.43 (0.16)	0.41 (0.11)	0.21	-6.65
**OCCIPITAL_INF_L**	0.32 (0.2)	0.29 (0.12)	0.2	-10.16
**OCCIPITAL_INF_R**	0.28 (0.22)	0.28 (0.15)	-0.01	0.6
**FUSIFORM_L**	0.11 (0.07)	0.1 (0.1)	0.19	-14.49
**FUSIFORM_R**	0.17 (0.1)	0.14 (0.09)	0.24	-13.24
**POSTCENTRAL_L**	0.34 (0.07)	0.34 (0.09)	-0.07	1.65
**POSTCENTRAL_R**	0.28 (0.11)	0.29 (0.09)	-0.12	4.44
**PARIETAL_SUP_L**	0.33 (0.12)	0.32 (0.17)	0.06	-2.52
**PARIETAL_SUP_R**	0.29 (0.18)	0.3 (0.15)	-0.08	4.55
**PARIETAL_INF_L**	0.53 (0.13)	0.57 (0.15)	-0.24	6.44
**PARIETAL_INF_R**	0.59 (0.14)	0.66 (0.14)	-0.46	11.11
**SUPRA_MARGINAL_L**	0.42 (0.08)	0.44 (0.13)	-0.21	5.33
**SUPRA_MARGINAL_R**	0.46 (0.08)	0.5 (0.09)	-0.38	7.04
**ANGULAR_L**	0.62 (0.18)	0.63 (0.18)	-0.08	2.29
**ANGULAR_R**	0.61 (0.15)	0.6 (0.12)	0.02	-0.37
**PRECUNEUS_L**	0.53 (0.1)	0.56 (0.13)	-0.29	6.22
**PRECUNEUS_R**	0.56 (0.11)	0.58 (0.09)	-0.2	3.56

**PARACENTRAL_LOBULE_L**	0.16 (0.13)	0.19 (0.15)	-0.21	18.97
**PARACENTRAL_LOBULE_R**	0.22 (0.15)	0.19 (0.11)	0.18	-10.83
**CAUDATENUCL_L**	0.14 (0.15)	0.13 (0.19)	0.04	-4.47
**CAUDATENUCL_R**	0.19 (0.16)	0.16 (0.2)	0.19	-17.23
**PUTAMEN_L**	0.47 (0.14)	0.49 (0.17)	-0.1	3.38
**PUTAMEN_R**	0.38 (0.18)	0.44 (0.23)	-0.29	15.7
**PALLIDUM_L**	-0.07 (0.12)	-0.06 (0.29)	-0.05	-16.74
**PALLIDUM_R**	0.06 (0.14)	0.06 (0.18)	-0.03	7.21
**THALAMUS_L**	0.18 (0.09)	0.21 (0.12)	-0.24	13.95
**THALAMUS_R**	0.22 (0.1)	0.25 (0.12)	-0.21	10.73
**HESCHL_L**	0.64 (0.15)	0.63 (0.17)	0.06	-1.45
**HESCHL_R**	0.76 (0.17)	0.8 (0.17)	-0.23	5.2
**TEMPORAL_SUP_L**	0.34 (0.08)	0.33 (0.11)	0.09	-2.61
**TEMPORAL_SUP_R**	0.36 (0.09)	0.37 (0.11)	-0.07	1.79
**TEMPORAL_POLE_SUP_L**	-0.12 (0.13)	-0.23 (0.17)	**0.72**	95.41
**TEMPORAL_POLE_SUP_R**	-0.1 (0.12)	-0.17 (0.1)	0.6	64.75
**TEMPORAL_MID_L**	0.41 (0.08)	0.4 (0.1)	0.06	-1.36
**TEMPORAL_MID_R**	0.43 (0.09)	0.43 (0.09)	0.02	-0.42
**TEMPORAL_POLE_MID_L**	-0.1 (0.11)	-0.12 (0.13)	0.19	22.73
**TEMPORAL_POLE_MID_R**	-0.01 (0.13)	-0.04 (0.14)	0.19	233.19
**TEMPORAL_INF_L**	0.27 (0.1)	0.25 (0.1)	0.15	-5.58
**TEMPORAL_INF_R**	0.29 (0.14)	0.26 (0.09)	0.21	-8.35
**CEREBELLUM_CRUS1_L**	0.17 (0.15)	0.16 (0.15)	0.1	-8.61
**CEREBELLUM_CRUS1_R**	0.12 (0.15)	0.12 (0.18)	0.04	-5.71
**CEREBELLUM_CRUS2_L**	0.22 (0.15)	0.22 (0.18)	0	-0.16
**CEREBELLUM_CRUS2_R**	0.13 (0.14)	0.17 (0.19)	-0.2	24.4
**CEREBELLUM3_L**	-0.37 (0.15)	-0.36 (0.15)	-0.08	-3.13
**CEREBELLUM3_R**	-0.4 (0.13)	-0.35 (0.14)	-0.32	-10.74
**CEREBELLUM45_L**	-0.14 (0.14)	-0.13 (0.12)	-0.13	-12.17
**CEREBELLUM45_R**	-0.08 (0.13)	-0.07 (0.1)	-0.09	-12.33
**CEREBELLUM6_L**	0.1 (0.14)	0.11 (0.14)	-0.11	15

**CEREBELLUM6_R**	0.11 (0.12)	0.14 (0.13)	-0.22	24.06
**CEREBELLUM7_L**	0.13 (0.21)	0.13 (0.17)	0	-0.32
**CEREBELLUM7_R**	0.12 (0.16)	0.16 (0.18)	-0.23	32.31
**CEREBELLUM8_L**	-0.03 (0.18)	-0.03 (0.17)	0.04	24.18
**CEREBELLUM8_R**	-0.04 (0.15)	-0.02 (0.18)	-0.1	-41.84
**CEREBELLUM9_L**	-0.06 (0.17)	-0.07 (0.16)	0.04	11.35
**CEREBELLUM9_R**	-0.04 (0.15)	-0.03 (0.17)	-0.08	-31.84
**CEREBELLUM10_L**	-0.52 (0.27)	-0.56 (0.18)	0.2	8.86
**CEREBELLUM10_R**	-0.48 (0.22)	-0.46 (0.21)	-0.1	-4.49
**VERMIS12**	-0.4 (0.15)	-0.36 (0.19)	-0.27	-11.49
**VERMIS3**	-0.18 (0.17)	-0.15 (0.15)	-0.16	-14.97
**VERMIS45**	-0.21 (0.16)	-0.17 (0.13)	-0.23	-16.26
**VERMIS6**	-0.14 (0.16)	-0.09 (0.16)	-0.32	-37.27
**VERMIS7**	-0.08 (0.17)	-0.02 (0.17)	-0.33	-69.4
**VERMIS8**	0.12 (0.19)	0.13 (0.18)	-0.04	6.51
**VERMIS9**	0.17 (0.2)	0.2 (0.2)	-0.13	14.48
**VERMIS10**	-0.39 (0.23)	-0.28 (0.19)	-0.53	-28.23
**MEDULLA**	-0.6 (0.12)	-0.62 (0.12)	0.18	3.59
**MIDBRAIN**	-0.23 (0.09)	-0.26 (0.09)	0.27	10.41
**PONS**	-0.47 (0.1)	-0.5 (0.1)	0.27	5.53
**WHITE_MATTER_L**	-0.41 (0.1)	-0.41 (0.13)	-0.05	-1.46
**WHITE_MATTER_R**	-0.39 (0.12)	-0.4 (0.13)	0.06	1.8
**CEREBELLUM_WM**	-0.29 (0.12)	-0.32 (0.14)	0.24	10.67

Supplementary table 5: Clusters with increased brain metabolism in SGCE mutation-positive compared to SGCE mutation-negative M-D patients (no decreases were found).

	MNI coordinates					
**Brain region**	**X**	**Y**	**Z**	**Zmax**	**Cluster size (mm3)[a]**	**P[b,c]**
Right precuneus	8	-48	16	4.56	1616	<0.001

	24	-54	22	3.82		
Left precuneus	-8	-62	34	3.72	360	<0.001
Left precuneus	-10	-58	16	3.60	48	<0.001
Right inferior parietal lobule	50	-52	42	3.58	88	<0.001
Right precuneus	16	-64	28	3.58	128	<0.001
Right precentral gyrus	20	-28	66	3.50	40	<0.001
Left middle occipital lobe	-40	-78	8	3.44	32	<0.001
Right lingual gyrus	16	-44	0	3.38	56	<0.001

Reported Z_{max} values have been adjusted for age and depression scores.

a based on p<0.001 uncorrected.

b uncorrected for multiple comparisons

c all results were not statistically significant after correction for multiple comparisons using either FDR and TFCE.

Supplementary table 6: FDG uptake associated with myoclonus dystonia CGI severity

	MNI coordinates					
**Brain region**	**X**	**Y**	**Z**	**Zmax**	**Cluster size (mm3)[a]**	**P[b,c]**
Right fusiform gyrus	26	-86	0	4.76	896	<0.001
Right inferior occipital lobe	36	-82	-4	4.01		
Right postcentral gyrus	52	-12	52	4.65	600	<0.001
Right supramarginal gyrus	52	-28	40	4.26	232	<0.001
Left middle occipital lobe	-34	-76	8	3.95	72	<0.001
Left superior temporal lobe	-46	-44	22	3.43	32	<0.001
Left supramarginal gyrus	-46	-46	24	3.40	16	<0.001
Right postcentral gyrus	44	-28	62	3.37	16	<0.001
Right postcentral gyrus	34	-30	60	3.37	8	<0.001
Right middle frontal lobe	30	42	18	-3.57	72	<0.001
Right cerebellum crus 1	52	-54	-30	-3.55	64	<0.001
Right caudate nucleus	12	14	6	-3.47	40	<0.001
Right superior frontal lobe	16	44	34	-3.33	16	<0.001

Reported Z_{max} values have been adjusted for age and depression scores

a based on p<0.001 uncorrected

b uncorrected for multiple comparisons

c all results were not statistically significant after correction for multiple comparisons using either FDR and TFCE.

In the link below the code used for voxel based analysis can be found.

https://github.com/jrdalenberg/FDG-PET-study-in-SGCE-positive-and-negative-Myoclonus-Dystonia
